# miRNA-23b as a biomarker of culture-positive neonatal sepsis

**DOI:** 10.1186/s10020-020-00217-8

**Published:** 2020-10-08

**Authors:** Ahlam Fatmi, Sid Ahmed Rebiahi, Nafissa Chabni, Hanane Zerrouki, Hafsa Azzaoui, Yamina Elhabiri, Souheila Benmassour, José Santiago Ibáñez-Cabellos, Mohammed Chems-Eddine Smahi, Mourad Aribi, José Luis García-Giménez, Federico V. Pallardó

**Affiliations:** 1 Laboratory of Applied Molecular Biology and Immunology, W0414100 Tlemcen, Algeria; 2 Laboratory of Microbiology Applied in Food, Biomedical and Environment, Tlemcen, Algeria; 3 Faculty of Medicine, Tlemcen Medical Centre University, 13000 Tlemcen, Algeria; 4 Neonatal Department of Specialized Maternal and Child Hospital of Tlemcen, 13000 Tlemcen, Algeria; 5 Center for Biomedical Network Research on Rare Diseases (CIBERER), Institute of Health Carlos III, Valencia, Spain; 6 INCLIVA Health Research Institute, Mixed Unit for rare diseases INCLIVA-CIPF, Valencia, Spain; 7 Department of Physiology, Faculty of Medicine and Dentistry, University of Valencia, Avenida Blasco Ibañez 15, 46010 Valencia, Spain

**Keywords:** Early-onset sepsis, Haemoculture, Late-onset sepsis, miR-23b, Newborns

## Abstract

**Background:**

Neonatal sepsis remains an important cause of morbidity and mortality. The ability to quickly and accurately diagnose neonatal sepsis based on clinical assessments and laboratory blood tests remains difficult, where haemoculture is the gold standard for detecting bacterial sepsis in blood culture. It is also very difficult to study because neonatal samples are lacking.

**Methods:**

Forty-eight newborns suspected of sepsis admitted to the Neonatology Department of the Mother-Child Specialized Hospital of Tlemcen. From each newborn, a minimum of 1–2 ml of blood was drawn by standard sterile procedures for blood culture. The miRNA-23b level in haemoculture was evaluated by RT-qPCR.

**Results:**

miR-23b levels increased in premature and full-term newborns in early onset sepsis (*p* < 0.001 and *p* < 0.005 respectively), but lowered in late onset sepsis in full-term neonates (*p* < 0.05) compared to the respective negative controls. miR-23b levels also increased in late sepsis in the negative versus early sepsis negative controls (*p* < 0.05). miR-23b levels significantly lowered in the newborns who died from both sepsis types (*p* < 0.0001 and *p* < 0.05 respectively). In early sepsis, miR-23b and death strongly and negatively correlated (correlation coefficient = − 0.96, *p* = 0.0019). In late sepsis, miRNA-23b and number of survivors (correlation coefficient = 0.70, *p* = 0.506) positively correlated.

**Conclusions:**

Lowering miR-23b levels is an important factor that favours sepsis development, which would confirm their vital protective role, and strongly suggest that they act as a good marker in molecular diagnosis and patient monitoring.

## Introduction

During the neonatal period, the immune system is still immature, and most immune responses are ensured by innate immunity, triggered following intimate contact between immune cells and microbes. In newborns, altered microbiota or microbial deprivation, as well as reduced microbial diversity, greatly increase the risk of immune dysregulation and proneness to inflammatory diseases. This makes neonates very fragile and more sensitive to several infectious diseases (Kumar and Bhat 2016; Lucignano et al. 2011).

Nowadays, neonatal sepsis is one of the most dangerous conditions to affect newborns during the first 28 days of life, and is a first-order public health system problem given its very high risk of mortality and morbidity (Bhandari 2014; Panwar et al. 2017). It is frequently divided into two types according to onset time: early-onset sepsis (EOS), when the process develops during the first 72 h of life; late-onset sepsis (LOS), when it occurs after the first 72 h. Unlike to fungi and parasites, bacteria and viruses are the commonest causative agents involved in neonatal sepsis aetiology (Ansari et al. 2015; Cortese et al. 2016).

Sepsis is commonly diagnosed by microbiological blood culture, but this can take days to perform, can suffer contamination and provide false-negative results. An empirical antibiotic therapy approach in neonatal sepsis is common clinical practice. In the presence of suspected bacterial infection, the use of random antibiotics is often unnecessary and prolongs the treatment of many uninfected newborns. Increasing the risk of multi-resistant strains emerging, however, or delaying or stopping antibiotic use in septicaemic newborns can also be catastrophic given rapid disease progression (Ng 2004; Shane et al. 2017). In addition, caring for newborns in specific hospital departments is a drain on human and financial resources (Atif et al. 2008; Wagstaff et al. 2019).

MicroRNAs (miRNAs), a class of small single-stranded non-coding regulatory RNAs of about 19 to 22 nucleotides, are involved in a wide range of biological processes and have opened a new window of hope to diagnose, and even treat, various diseases. miRNA binds to specific mRNA molecules to inhibit the expression of target genes or to degrade mRNA, which then contributes to cell proliferation, differentiation, development, metabolism, apoptosis and other physiological activities (Wu et al. 2015; Lenkala et al. 2014). Given their role in various cellular processes, recent studies reveal how miRNAs may have the potential to be an early biomarker in a number of diseases, including sepsis (Wang et al. 2010). Among microRNAs, we can identify miR-23b. Chromosomal region 9q22.32 produces miR-23b. The combined body of available works suggests that miR-23b expression is not only modulated by a diverse array of stimuli in cells from different lineages, but also participates in multiple gene regulatory feedback loops (Wang et al. 2018). Nevertheless, the role of miRNAs in neonatal sepsis has not been widely explored. It is noteworthy that miR-23b is a proven and important regulator of the innate immune response in both cancer and several inflammatory processes (Zhu et al. 2012). In addition, some studies show that microR-23b expression in the peripheral blood of sepsis patients is related to the manifestation of an inflammatory state and may, therefore, be used to evaluate the severity and prognosis of adults patients with this disease (Ou et al. 2018). In another study, miR-23b is proposed as an essential contributor to cardiac fibrosis activation to mediate the development of myocardial dysfunction in late sepsis. This report suggests that blocking miR-23b expression might be an effective approach to prevent sepsis-induced cardiac dysfunction (Zhang et al. 2018). Another study reveals that miR-23b inhibition down-regulates the expression of programmed death ligand-1 (PD-L1) on splenic T lymphocytes of septic mice. This discovery opens up new therapeutic pathways in late stages of the septic phase (Beltrán-García et al. 2020). Finally, another study demonstrates that miR-23b is an anti-inflammatory factor that negatively regulates the inflammatory responses induced by lipopolysaccharide (LPS) by targeting metalloproteinase 10 (ADAM10) (Zhang et al. 2019a).

Haemocultures are the “gold standard” for identifying bacterial and fungal infections in the bloodstream. However, they are limited by large volume requirements to maximise sensitivity and often imply long incubation times. To address some of these limitations, many advances have been developed to improve sensitivity and to reduce the time required to identify the cause of bloodstream infections. Molecular amplification techniques have been developed to replace the incubation step in blood culture targeting conserved regions of microbial genomes for amplification, such as rRNA genes and interspace region 16S–23S (Tsalik et al. 2010; Gurtler and Stanisich 1996; Draz et al. 2013).

In this study, we attempted to estimate the expression levels of candidate circulating miR-23b in small cohorts of newborns diagnosed with early (EOS) or late (LOS) sepsis by microbiological blood culture test in the Neonatology Department of Mother & Child Specialized Hospital Establishment of Tlemcen (northwest Algeria). We show for the first time that miR-23b can be considered a potential marker of sepsis in haemocultures from neonate peripheral blood samples. Hence, we demonstrated that miR-23b levels increased in EOS, but lowered in LOS, compared to the respective negative controls. These levels also increased in the LOS negative controls compared to the EOS negative controls. Therefore, the drop in miR-23b levels would undoubtedly be an important factor that favours sepsis development, which would confirm their vital protective role on the one hand, and would strongly suggest their use as a good marker in both molecular diagnosis and patient monitoring on the other hand.

## Patients and methods

### Ethical aspects

The present study was approved by the Local Ethics Committee of Tlemcen University. Parents or legal guardians gave written informed consent so that the samples from all the participating infants could be used according to the Declaration of Helsinki.

### Study population

Of the 2561 newborns admitted during a 12-month period to the Neonatology Department of Mother & Child Specialized Hospital Establishment (EHS, *Etablissement Hospitalier Spécialisé Mère-Enfant*) of Tlemcen (northwest Algeria), 254 (9.91%) newborns with sepsis were recorded. Forty-eight cases aged up to 28 days with clinical features of sepsis (e.g. fever, respiratory distress, bradycardia, tachycardia, convulsions, cyanosis), and an association, or not, with premature rupture of membranes (PROM), and abnormal amniotic liquid as risk factors (Singer et al. 2016), who met the neonatal sepsis inclusion criteria, were recruited in a prospective cohort study. The exclusion criteria included those patients without sepsis clinical features or those who had received antibiotherapy before sampling. Newborns included 20 females and 28 males. The 48 patients were randomly divided into two groups of 27 EOS, including nine cases of preterm newborns and 21 LOS patients.

### Samples for blood haemoculture

Peripheral blood samples (1–2 mL) were inoculated into aerobic bottles containing paediatric haemoculture medium (BIOSCAN, Sétif, Algeria) to be incubated at 37 °C for 4–6 h with agitation. Aliquots of 2 mL were collected and stored at − 80 °C until RNA extraction.

Total RNA extraction, including miRNA, was performed with a minimum of 200 μL of cell-free supernatant obtained after centrifuging an aliquot at 1200 rpm for 10 min using the miRNeasy Serum/Plasma kit (Qiagen, Valencia, Spain) according to the manufacturer’s protocol. RNA was eluted with 20 μL of RNase-free water and was then quantified in a NanoDrop ND 2000 UV spectrophotometer (Thermo Scientific, Wilmington, DE, USA).

### Reverse transcription PCR and real-time qPCR

Total RNA (1 μL) was converted into complementary DNA (cDNA) by reverse transcriptase using the miRNA TaqMan reverse transcription kit and miRNA-specific stem and loop primers (Part No. 4366597, Applied Biosystems. Inc., CA, USA). Real-time PCR was performed in an Applied BioSystem 7900HT Thermocycler (Applied Biosystems/Thermo Fisher, USA) with 40 cycles. The primers herein used were designed for miRNA-23b (hsa-miR-23b (Assay ID 002126). Thermo Fisher, CA, USA), and U6 snRNA (U6-snRNA (Assay ID 001973). Thermo Fisher, CA, USA), was used for standardisation purposes by the delta-delta CT method (2-^ΔΔCT^).

### Statistical analysis

The results represent the mean (± standard deviation) of the median values of three independent replicate experiments. Analyses of variance were carried out by Mann Whitney *U* or Kruskal–Wallis non-parametric tests using GraphPad Prism 8.0.1 (244) as data were not normally distributed (Olsen 2003). *P*-value < 0.05 was considered statistically significant.

## Results and discussion

Forty-eight haemocultures were performed in this study, of which 18.75% were premature and 81.25% at-term. Gender, temperature, heart rate, respiratory rate, glycaemia and caesarean vs. vaginal delivery characteristics were not statistically different between the control and the positive haemoculture groups. However, neonates’ weight in both sepsis types was significantly different to C-reactive protein (CRP), which significantly differed in the EOS neonates. The clinical information of the 48 patients is shown in Table 1.

**Table 1:** Characteristics of the newborn patients with sepsis in the present study

EOS/LOS (***n*** = 27, 21)	Full-term patients				Premature patients			***p***
	Co/NH (control) (9, 6)	SP/PH (7, 12)	DP/PH (2, 1)	DP/NH (0, 2)	Co/NH (control) (4, 0)	SP/PH (2, 0)	DP/PH (3, 0)	
**Gender** (M/F)								
EOS	5/4	4/3	1/1	–	1/3	1/1	2/1	NS
LOS	6/0	6/6	0/1	2/0	–	–	–	NS
**Weight** (kg)								
EOS	2.87 ± 0.76	3.16 ± 0.65	3.30 ± 0.14	–	2.01 ± 0.09	1.43 ± 0.23	1.63 ± 0.57	< 0.001
LOS	3.17 ± 0.86	3 ± 0.86	2.5 ± 0	2.9 ± 0.28	–	–	–	< 0.001
**T** (° C)								
EOS	36.17 ± 1.43	35.05 ± 1.42	34.5 ± 1.27	–	36.35 ± 2.30	33.07 ± 2.21	36.17 ± 1.20	NS
LOS	37.33 ± 2.85	38.3 ± 1.53	37.4 ± 0	39.05 ± 0.95	–	–	–	NS
**HR** (BPM)				–				
EOS	136 ± 16.37	143.6 ± 17.87	125 ± 9.9	–	120.5 ± 19.58	125 ± 7.07	150 ± 10	NS
LOS	151.8 ± 27	140.3 ± 17.27	180 ± 0	135 ± 7.07	–	–	–	NS
**RR** (BrPM)				–				
EOS	55.3 ± 14.56	55.7 ± 17.1	38 ± 5.66		58 ± 5.42	42 ± 2.83	62.67 ± 4.72	NS
LOS	50.7 ± 14.01	52.5 ± 6.1	52 ± 0	42 ± 2.83	–	–	–	NS
**Gly** (mg/dL)								
EOS	0.95 ± 0.48	0.63 ± 0.23	0.52 ± 0.60		0.73 ± 0.25	–	0.45 ± 0.15	NS
LOS	1.12 ± 0.22	0.65 ± 0.21	–	0.66 ± 0.17	–	–	–	NS
**CRP** (mg/dL)								
EOS	25.78 ± 17.87	41.17 ± 30.75	–	–	63 ± 88.25	–	–	< 0.0001
LOS	47 ± 81.37	39.87 ± 29.78	–	42 ± 25.45	–	–	–	NS
**VD vs. CD**				–			–	
EOS	5/4	5/2	1/1	–	2/2	1/1	1/2	NS
LOS	5/1	1/1	1/0	2/0	–	–	–	NS

Data are presented as the mean ± standard deviation (*X* ± SD)

*BPM* beats per minute, *BrPM* breaths per minute, *CF* Cardiac frequency, *Co/ NH* control newborns with negative haemoculture, *CRP* C-reactive protein, *DP/NH* patients who died with negative haemoculture, *DP/PH* patients who died with positive haemoculture, *EOS* early onset sepsis, *F* female, *Gly* glycaemia, *HR* Heart rate, *EOS* early onset sepsis, *LOS* late onset sepsis, *M* male, *NS* not significant, *RR* Respiratory rate, *SP/PH* patients who survived with positive haemoculture, *VD vs. CD* vaginal vs. caesarean delivery

### Changes in the miRNA-23b expression levels in early onset sepsis

**Fig. 1:**
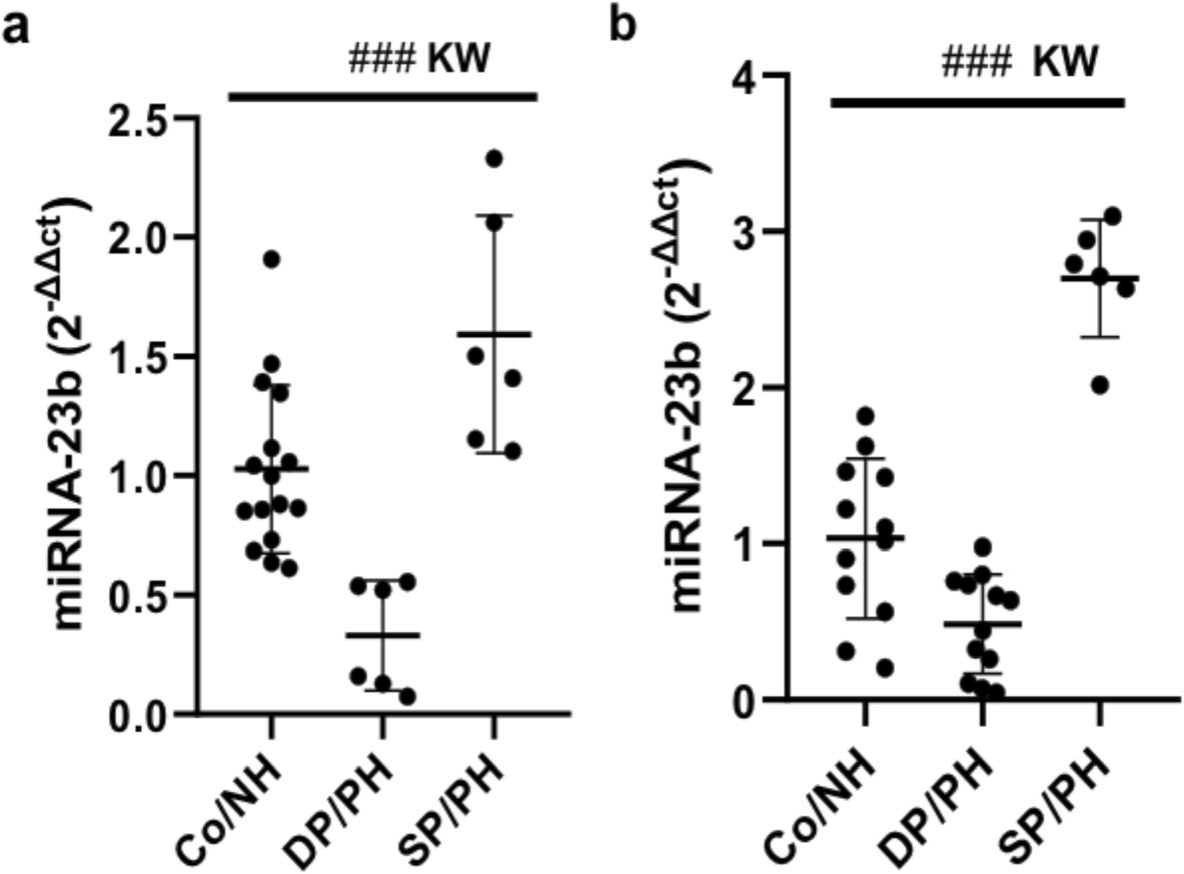
Changes in the miRNA-23b expression levels in early onset sepsis. Scatter plot values a on the left represent the miR-23b level for EOS in the at-term newborns, measured by (2^-ΔΔCT^). While that scatter plot in b on the right represent the miR-23b level in the premature newborns. The line inside the boxes corresponds to the median values. For the at-term patients, Co/NH (*n* = 9), DP/PH (*n* = 2), SP/PH (*n* = 7). For the premature patients, Co/NH (*n* = 4), DP/PH (*n* = 3), SP/PH (*n* = 2). Co/NH: negative haemoculture, DP/PH: positive haemoculture in dead newborns, SP/PH: positive haemoculture in the newborns who survived. KW: Kruskal-Wallis. The sharp indicate significant differences highlighted between all groups using the Kruskal-Wallis test: ### *p* < 0.001

The miR-23b expression levels in the neonatal sepsis samples were analysed by the quantitative real-time PCR method. Our results showed that, compared to the control group, the miR-23b expression levels significantly differed in the neonatal sepsis samples either in the at-term or premature neonates (*p* < 0.001 KW) (Fig. 1). The miR-23b expression significantly lowered in the neonates who died of sepsis (*p* < 0.0001, *p* < 0.05 at-term and premature infants, respectively), and significantly increased in the neonates who survived with a positive haemoculture (*p* < 0.005, *p* < 0.001). These results reveal that miR-23b expression correlates with sepsis progression.

### Changes in the miR-23b expression levels in late onset sepsis

**Fig. 2:**
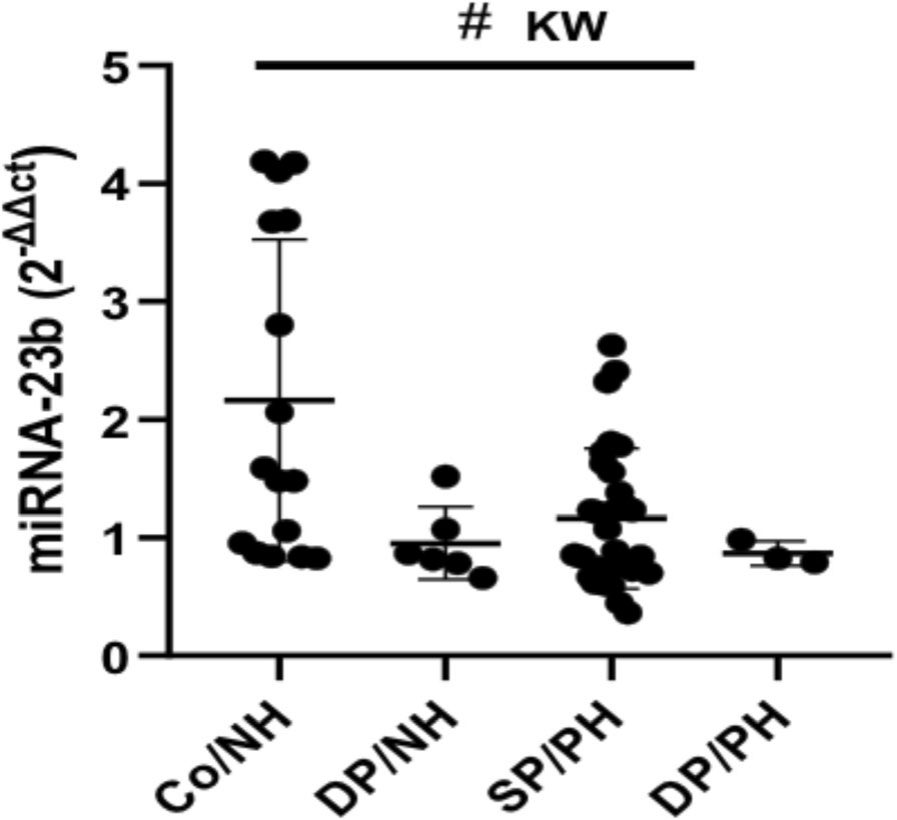
Changes in the miR-23b expression levels in late onset sepsis. Scatter plot values represent the miR-23b level for LOS in at-term newborns, measured by (2^-ΔΔCT^). The line inside the boxes corresponds to the median values. Co/NH (*n* = 6), DP/NH (*n* = 2), SP/PH (*n* = 12), DP/PH (*n* = 1). Co/NH: negative haemoculture, DP/NH: negative haemoculture in the dead newborns, DP/PH: positive haemoculture in the newborns who died, SP/PH: positive haemoculture in the newborns who survived, KW: Kruskal-Wallis. The sharp indicate significant differences highlighted between all groups using the Kruskal-Wallis test: # *p* < 0.05

Figure 2 shows how the miR-23b expression in LOS significantly lowers in both the dead and surviving newborns with a positive haemoculture, with *p* < 0.005 and *p* < 0.05, respectively, compared to the controls and for all comparisons (*p* < 0.05 KW). Two cases presenting the clinical signs of sepsis died, but the haemoculture was negative. In this case, we recorded a significant drop in the miR-23b level with a negative haemoculture (*p* < 0.05). This case was considered a false-negative haemoculture, probably due to the sampling time (Hall and Lyman 2006) or another limitation, like the presence of unculturable or fastidious microorganisms that could decrease its sensitivity (Jordana-Lluch et al. 2014).

### Change in the miRNA-23b expression levels in newborns in two different stages

**Fig. 3:**
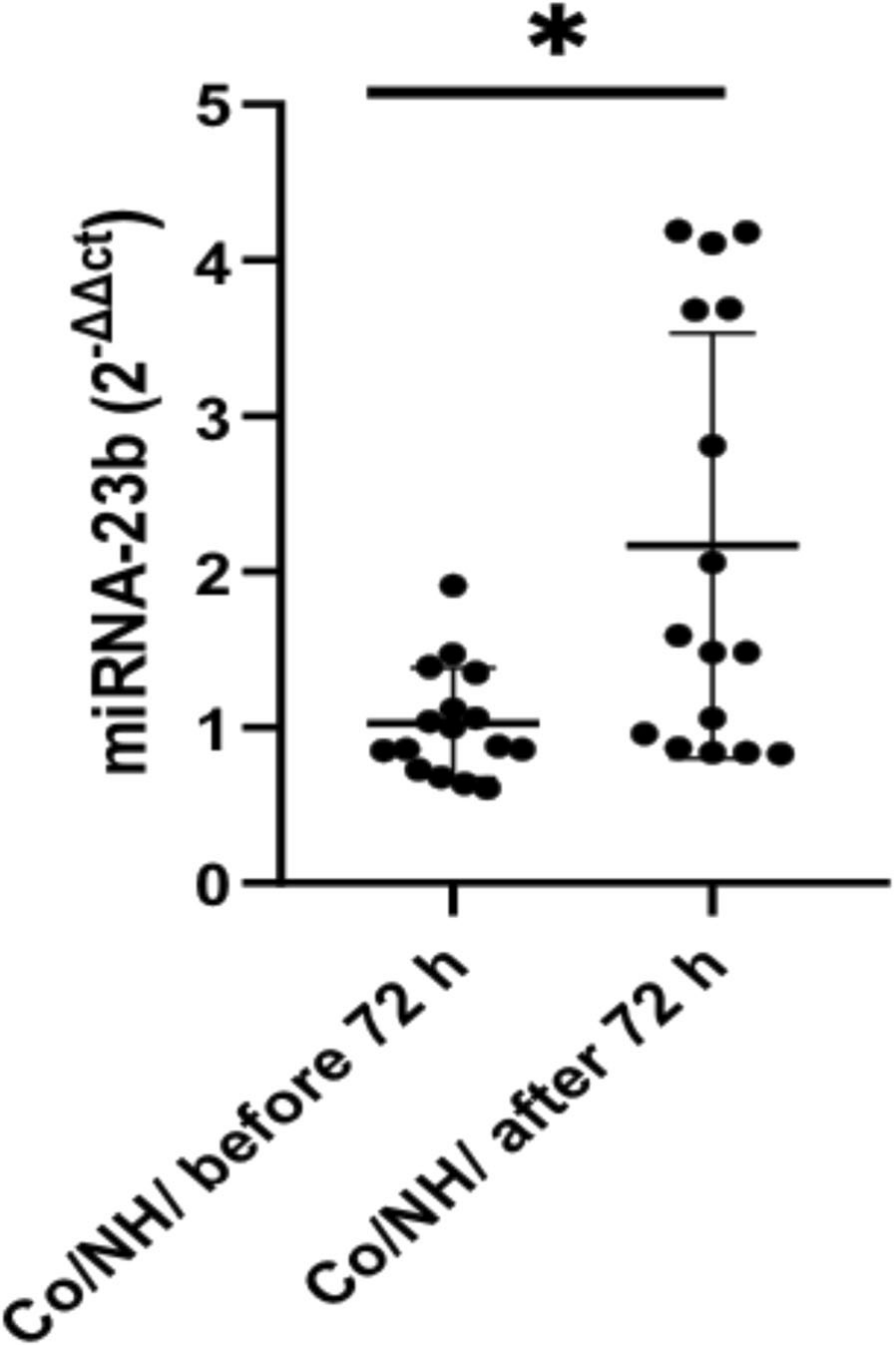
Change in the miRNA-23b expression levels in newborns at two different stages. Scatter plot values represent the miR-23b level for the negative control of EOS (*n* = 9) and the negative control of LOS (*n* = 6) in terms of the newborns, measured by (2^-ΔΔCT^). The line inside the boxes corresponds to the median values. LOS/ Bac-: negative haemoculture in LOS, Co/NH before 72 h: negative haemoculture in EOS, where patients’ age was less than 72 h, Co/NH / before 72 h: negative haemoculture in LOS, where patients’ age was more than 72 h. *p = 0.011 via the Mann-Whitney *U* test

The differences between our results when comparing EOS and LOS led us to think back to the starting point before sepsis appeared. Figure 3 shows the miR-23b expression level after the first 72 h of life and beyond that time in the control patients. The results revealed a significant increase in the miR-23b level after 72 h of live performance *p* < 0.05 compared to that before the first 72 h of life.

### The correlation between miR-23b, sepsis and death during the neonatal period

**Fig. 4:**
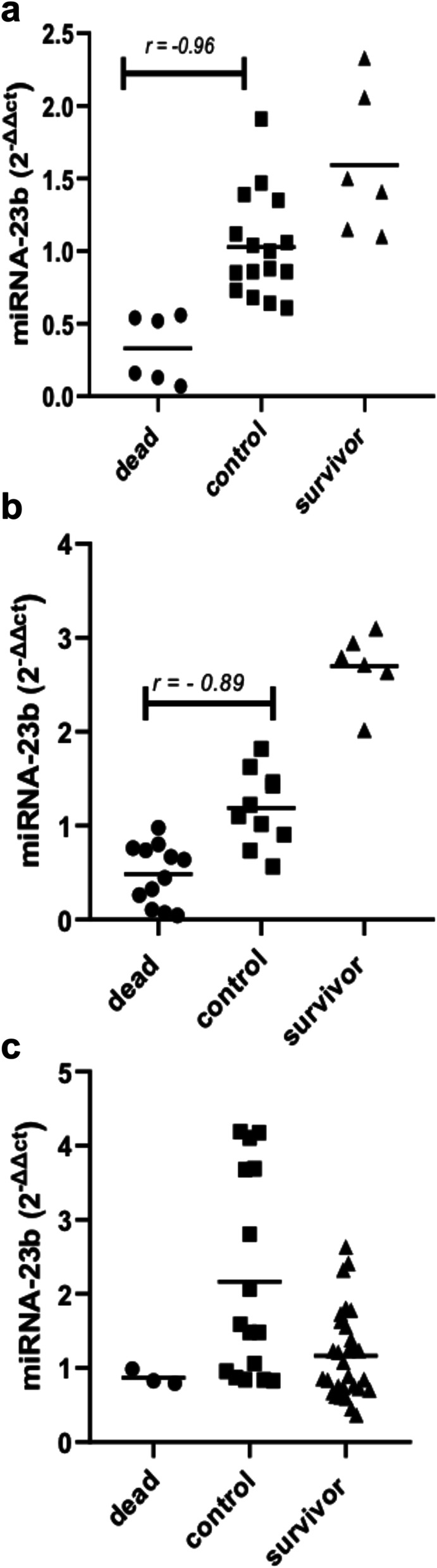
Correlation between miRNA-23b expression and dead newborns with sepsis. Individual values represent the correlation with the miR-23b level, survivor and dead in newborns with sepsis. **a**: in early onset sepsis in at-term newborns, **b**: early onset sepsis in premature newborns, **c**: late onset sepsis in at-term newborns. Control: patients with negative haemoculture, dead: dead patients with sepsis, survivor: survivor patients with sepsis, *r*: correlation coefficient

Figure 4 shows a very strong negative correlation between miR-23b and death in early sepsis patients (*r* = − 0.96, *p* = 0.002), the same for premature, with a negative correlation with miR-23b and death sepsis (*r* = − 0.89, *p* = 0.0001), but the miR-23b level correlated negatively with sepsis (*r* = − 0.81, *p* = 0.39). With late sepsis, a low negative non-significant correlation is observed between miR-23b and the appearance of sepsis (*r* = − 0.26, *p* = 0.32). Nevertheless, we show a positive correlation between miRNA-23b levels in both the control and non-survivor patients (*r* = 0.70, *p* = 0.506).

Infant and late foetal deaths are key factors when assessing a country’s level of social protection (Say et al. 2009; Gonzalez and Gilleskie 2017). In neonatal sepsis, the leading treatment is antibiotics, which target the infecting pathogen, but not the inflammatory process that continues to increase. Therefore, an ideal treatment approach should include antimicrobial and anti-inflammatory drugs to neutralise the rising inflammatory cascade and the resulting “cytokine storm” in neonatal sepsis (Nedeva et al. 2019). Newborns immune status during the perinatal period differs from that of adults. Neonatal immune responses are generally directed against the generation of T helper type 1 (Th1)-related proinflammatory immune responses, while favouring the Th2-related anti-inflammatory /immunosuppressive response (Kollmann et al. 2012). This process represents an efficient strategy to address the unique challenges of the neonatal period, including maintaining tolerance to maternal antigens in utero and balancing the transition from the sterile intrauterine environment to the antigen-rich outside world (Kollmann et al. 2012).

miRNAs are endogenous, non-coding, single-stranded RNAs (~ 22 nucleotides long) with the ability to degrade mRNA or inhibit translation, which then regulates gene expression at the post-transcriptional level (Wu et al. 2015). We know that the expression of ≥30% of human genes is controlled by miRNAs (Bartel 2004). miRNAs also regulate molecular signalling pathways and immune activities (Yu et al. 2018). The invasion of pathogenic microorganisms, followed by rapid miRNAs production, promote the release of inflammatory factors that cause immune hyperactivity, and induce apoptosis or degrading inflammatory factors that can provoke immunosuppression (Li et al. 2014; Chen et al. 2013).

The biomarkers frequently used in neonatal sepsis are still not completely conclusive. But have shown some potential for in vitro diagnoses (Kingsley Manoj Kumar and Vishnu Bhat 2015). Since their discovery, circulating miRNAs in human peripheral serum are used as biomarkers of various cancer types. The use of miRNAs as diagnostic and prognostic markers has extended to other diseases, including sepsis, but their role in infectious diseases has rarely been studied (Wang et al. 2013; Wang et al. 2012). One of the main obstacles to establish a well-defined link between miRNAs and sepsis lies in the fact that sepsis can be caused by very different factors that cause similarities and differences, which influence the patient’s situation itself and make sepsis so very complicated (Stearns-Kurosawa et al. 2011). This is why the association of miRNAs with sepsis diagnosis remains controversial (Zhang et al. 2019b).

miRNAs from different biological fluids can be used for the early prediction and evaluation of neonatal sepsis, where various miRNAs are down-regulated, and contribute to the initiation of the immune response to infection (Chen et al. 2014). To date, no studies are available on miRNAs in both neonatal sepsis types, i.e., EOS and LOS. To the best of our knowledge, no study has investigated miRNAs expression levels in haemocultures from septic patients and their change according to neonatal sepsis types.

The neonatal immune response to sepsis depends on the timing of onset, relative pathogens and developmental age (Sweeney et al. 2018), and is markedly different from the immune response in adults because of specific neonatal microbial susceptibility and atopic properties. Differences have been reported in the regulation of target gene expression by miRNAs in innate immunity (Yu et al. 2018). A study of ten immune-regulating miRNAs, whose expression significantly altered more than 2-fold in neonates with sepsis compared to uninfected neonates, showed that miRNA expression levels were altered, and that this alteration in miRNAs modulated the immune response during neonatal sepsis so as to represses inflammatory response (Chen et al. 2014). In another study (Cheng et al. 2018), low miRNA-26a levels have been correlated with the up-regulation of IL-6 expression in blood mononuclear cells and serum. Nevertheless, neither newborns age nor sepsis type has been specified. There are also reports indicating that miR-15a/16 can be used as a potential biomarker for the diagnosis and prognosis of neonatal sepsis, and that miRNA15a/16 regulation may limit the inflammatory response to LPS (Wang et al. 2015).

Although the discovery of miR-23b is recent (Wu et al. 2015; Ou et al. 2018), intense research efforts have been made to show that it is involved in various physiological and pathophysiological processes (Wang et al. 2018). So, it has been revealed as an essential moderator of several physiological pathways that regulate the differentiation of many cell lines, such as keratinocytes, chondrocytes and skeletal muscle. miR-23b also regulates inflammatory response in several autoimmune diseases through suppressing proinflammatory signalling pathways in resident cells, such as human fibroblast-likesynoviocytes, and in primary kidney cells and astrocytes from mice (Bordon 2012). miR-23b also plays a critical role in certain pathologies, including acute myocardial infarction, inflammatory heart diseases and sepsis-induced cardiac dysfunction (Grossi et al. 2018; Zhao et al. 2016), diabetic nephropathy (Zhao et al. 2016) and prostate cancer (Pimenta et al. 2018). We herein demonstrate for the first time the presence of miR-23b in haemocultures from neonatal sepsis and their interest for diagnosis and prognosis in early and late sepsis.

In sepsis, miR-23b has been reported to be down-regulated in peripheral blood mononuclear cells (PBMCs) from adult patients and in the LPS-induced THP-1 human monocytic cell line, and has been negatively correlated with the production of proinflammatory cytokines. Increased miR-23b expression has been shown to induce the down-regulation of proinflammatory cytokines production and LPS-stimulated apoptosis (Zhang et al. 2019a).

In the present study, we revealed that at-term birth, the neonates with negative haemocultures (the control group) presented low miR-23b levels during the first 72 h of life, which started to increase after 72 h. Conversely, the neonates with a positive haemoculture who did not survive infection always showed the lowest miR-23b levels, regardless of whether they were premature or born at term. In addition, the neonates with a positive haemoculture who survived infection had high miR-23b levels during the first 72 h of life, irrespectively of whether they were born at term or premature. Thus the increase in miR-23b levels during the first 72 h of life in septic neonates may be a potent prognostic factor for survival and a sensitive clinical marker in both preterm and at-term neonates.

It has been recently shown with an animal model of sepsis that miRNA profiles in CD8^+^ T cells from adult and neonatal mice were surprisingly similar during infection, but infection miRNA levels differed when it was absent. In particular, marked differences were observed in the miR-29 and miR-130 expression levels between adult and neonatal cells before infection. Likewise, changes in the expression of messenger RNA targets have been noted for both miR-29 and miR-130 (Wissink et al. 2015).

Our study indicated a difference in the miR-23b expression levels in both EOS and LOS. The miR-23b expression levels increased in the EOS patients with a positive haemoculture and lowered in the LOS patients with either premature or full-term newborns. Nevertheless, we also observed a difference in expression over time in the control group before and after 72 h of birth. This could be due to differences in the genome expression patterns in newborns between EOS and LOS. Exclusively to newborns, uninfected status and host response to sepsis are significantly affected by time of birth (Wynn et al. 2015; Raymond et al. 2017), in which immune system development is a continuous process throughout embryogenesis and into childhood. Hence the different miRNAs expression in neonatal sepsis could be considered a developmental characteristic of the immune response (Chen et al. 2014). Early and late sepsis responses considerably differ depending on the postnatal age at the time of sepsis (Ng et al. 2018). By controlling postnatal age in studies on epigenetic changes during neonatal sepsis, we were able to better understand the immune mechanism in newborns and to identify therapeutic targets. From these results, we suggest the possibility of using miR-23b levels as an in vitro diagnosis marker, which can be used to differentiate between EOS and LOS. miR-23b levels are up-regulated during the first 72 h of life and down-regulated over time during this period.

## Conclusions

In this first report, we demonstrate the usefulness of miRNAs in haemocultures from neonates, and the role of miR-23b as a potent biomarker in sepsis. This study could be of much interest in not only research, but also in Translational Medicine, and more specifically in Neonatal Infectiology, where the use of large volumes of blood is not possible. *In fine*, this study provides additional elements into the molecular approach for diagnosing and treating neonatal sepsis. These elements include three essential points: (i) miR-23b plays a vital role in neonatal sepsis; (ii) the expression of miR-23b differs during the neonatal period; (iii) miR-23b expression levels are up-regulated in EOS and down-regulated in LOS.

## Data Availability

No applicable.

## Abbreviations

BPM: Beats per minute
BrPM: Breaths per minute
CF: Cardiac frequency
Co/NH: Control newborns with negative haemoculture
CRP: C-reactive protein
DP/NH: Patients who died with negative haemoculture
DP/PH: Patients who died with positive haemoculture
EOS: Early-onset sepsis
F: Female
Gly: Glycaemia
HR: Heart rate
KW: Kruskal-Wallis
LOS: Late-onset sepsis
LPS: Lipopolysaccharide
M: Male
miRNAs: MicroRNAs
NH: Negative haemoculture
NS: Not significant
PBMCs: Peripheral blood mononuclear cells
PROM: Premature rupture of membranes
PD-L1: Programmed death ligand-1
RR: Respiratory rate
SP/PH: Patients who survived with positive haemoculture
VD vs. CD: Vaginal vs. caesarean delivery
