# Correction to: miRNA-23b as a biomarker of culture-positive neonatal sepsis

**DOI:** 10.1186/s10020-020-00257-0

**Published:** 2020-12-14

**Authors:** Ahlam Fatmi, Sid Ahmed Rebiahi, Nafissa Chabni, Hanane Zerrouki, Hafsa Azzaoui, Yamina Elhabiri, Souheila Benmansour, José Santiago Ibáñez-Cabellos, Mohammed Chems-Eddine Smahi, Mourad Aribi, José Luis García-Giménez, Federico V. Pallardó

**Affiliations:** 1 Laboratory of Applied Molecular Biology and Immunology, W0414100 Tlemcen, Algeria; 2 Laboratory of Microbiology Applied in Food, Biomedical and Environment, Tlemcen, Algeria; 3 Faculty of Medicine, Tlemcen Medical Centre University, 13000 Tlemcen, Algeria; 4 Neonatal Department of Specialized Maternal and Child Hospital of Tlemcen, 13000 Tlemcen, Algeria; 5 Center for Biomedical Network Research on Rare Diseases (CIBERER), Institute of Health Carlos III, Valencia, Spain; 6 Mixed Unit for Rare Diseases INCLIVA-CIPF, INCLIVA Health Research Institute, Valencia, Spain; 7 Department of Physiology, Faculty of Medicine and Dentistry, University of Valencia, Avenida Blasco Ibañez 15, 46010 Valencia, Spain

## Correction to: Molecular Medicine (2020) 26:94 https://doi.org/10.1186/s10020-020-00217-8

Following publication of the original article (Fanti et al. 2020), the authors identified an error in the author name of Souheila Benmansour.

The incorrect author name is: Souheila Benmassour.

The correct author name is: Souheila Benmansour.

Also, the authors Souheila Benmansour and Mohammed Chems-Eddine Smahi were affiliated incorrectly.

The correct affiliation for Souheila Benmansour is:

^1^Laboratory of Applied Molecular Biology and Immunology, W0414100 Tlemcen, Algeria.

^4^Neonatal Department of Specialized Maternal and Child Hospital of Tlemcen, 13,000 Tlemcen, Algeria.

The correct affiliation for Mohammed Chems-Eddine Smahi is:

^1^Laboratory of Applied Molecular Biology and Immunology, W0414100 Tlemcen, Algeria.

^4^Neonatal Department of Specialized Maternal and Child Hospital of Tlemcen, 13,000 Tlemcen, Algeria.

The original article has been corrected.
